# Comparative analysis of miRNA expression during the development of insects of different metamorphosis modes and germ-band types

**DOI:** 10.1186/s12864-017-4177-5

**Published:** 2017-10-11

**Authors:** Guillem Ylla, Maria-Dolors Piulachs, Xavier Belles

**Affiliations:** Institute of Evolutionary Biology, CSIC-Universitat Pompeu Fabra, Passeig Marítim 37, 08003 Barcelona, Spain

**Keywords:** miRNAs, Embryogenesis, Maternal-to-zygotic transition, Germ-band, Metamorphosis, *Blattella*, *Tribolium*, *Drosophila*

## Abstract

**Background:**

Do miRNAs contribute to specify the germ-band type and the body structure in the insect embryo? Our goal was to address that issue by studying the changes in miRNA expression along the ontogeny of the German cockroach *Blattella germanica*, which is a short germ-band and hemimetabolan species.

**Results:**

We sequenced small RNA libraries representing 11 developmental stages of *B. germanica* ontogeny (with especial emphasis on embryogenesis) and the changes in miRNA expression were examined. Data were compared with equivalent data for two long germ-band holometabolan species *Drosophila melanogaster* and *Drosophila virilis*, and the short germ-band holometabolan species *Tribolium castaneum*. The identification of *B. germanica* embryo small RNA sequences unveiled miRNAs not detected in previous studies, such as those of the MIR-309 family and 54 novel miRNAs. Four main waves of miRNA expression were recognized (with most miRNA changes occurring during the embryonic stages): the first from day 0 to day 1 of embryogenesis, the second during mid-embryogenesis (days 0–6), the third (with an acute expression peak) on day 2 of embryonic development, and the fourth during post-embryonic development. The second wave defined the boundaries of maternal-to-zygotic transition, with maternal mRNAs being cleared, presumably by Mir-309 and associated scavenger miRNAs.

**Conclusion:**

miRNAs follow well-defined patterns of expression over hemimetabolan ontogeny, patterns that are more diverse during embryonic development than during the nymphal stages. The results suggest that miRNAs play important roles in the developmental transitions between the embryonic stages of development (starting with maternal loading), during which they might influence the germ-band type and metamorphosis mode.

**Electronic supplementary material:**

The online version of this article (10.1186/s12864-017-4177-5) contains supplementary material, which is available to authorized users.

## Background

Do embryonic miRNAs influence the germ-band type and metamorphosis mode of insects? Long germ-band insects, such as the fruit fly *Drosophila melanogaster*, form most of their segments simultaneously at the blastoderm stage, i.e., before gastrulation. In contrast, short germ-band species start gastrulation with just a few segments, and then progressively add new ones from an undifferentiated growth zone situated at the posterior end of the embryo [1]. Short germ-band development is typical of basal insects such as locusts and cockroaches, whereas more derived species such as those of the genus *Drosophila* predominantly undergo long germ-band development [2]. The differences between these two developmental extremes are paralleled by the differential expressions of patterning genes [3, 4]. In general, the participation of miRNAs in gene-regulatory networks is crucial, increasing the precision of expression and reducing noise [5–9]. It is therefore likely that miRNAs influence the mechanisms that specify the germ-band type and body structure, which is related to the metamorphosis mode, in insect embryos.

We examined the changes in miRNA expression over the ontogeny of the German cockroach *Blattella germanica*, using 11 small RNA libraries prepared and sequenced in our laboratory, representing developmentally important stages. The basic details of the miRNA complement of *B. germanica*, a short germ-band, hemimetabolan species, are known [10, 11], as are a number of miRNA functions, especially in postembryonic development [12–15]. In this work, the small RNA libraries of *B. germanica* early embryo were compared with those previously published of two long germ-band holometabolan species, the flies *D. melanogaster* and *Drosophila virilis*, and the red flour beetle *Tribolium castaneum*, a short germ-band holometabolan species [16, 17]. The distinction between the hemimetabolan and holometabolan modes of metamorphosis is important: hemimetabolan species develop the basic adult body structure during embryogenesis, whereas holometabolans delay this until the pupal stage [18, 19]. miRNA commonalities between *B. germanica* and *T. castaneum*, as opposed to those of the two *Drosophila* species studied, were assumed to be associated with the germ-band type, whereas those of *T. castaneum* and the *Drosophila* species, as opposed to *B. germanica*, were assumed to be associated with the mode of metamorphosis followed.

## Results

### Novel miRNAs in the cockroach embryo

*B. germanica* embryogenesis was completed in 18 days at the set temperature of 29 °C. Nymph development in colony reared at this temperature lasted between 22 and 24 days and comprised six instars. Eleven stages covering essential developmental moments and characteristic hormonal contexts (Table 1) were chosen for examination. Two libraries of small RNAs were prepared for each examined stage and sequenced using Illumina NextSeq technology. This provided 292 million paired end reads, with each library having 9.2–17 million read pairs. After removing adapters, filtering out low quality reads, and merging read pairs, 148,674,256 million reads were mapped to the *B. germanica* genome and used in analyses (Additional file 1: Table S1).

**Table 1:** Biological data corresponding to the 11 small RNA libraries used in the present work

Library	Developmental period	Age (AO: after oviposition)	Percentage of embryo development (ED)	Embryo development and Tanaka (1976) stage	Hormonal context
NFE	Non-fertilized egg	Day 8 of the first gonadotrophic cycle (in preoviposition)	**–**	**--**	Not determined within the egg. High levels of 20E and JH in the surrounding haemolymph.
ED0	Embryo	8 h AO, when the ootheca is still vertical	2%.	Only yolk granules observed.	No detectable levels of 20E and JH
ED1	Embryo	24 h AO	6%	Energids at low density spread among the yolk granules. Tanaka stage 1	No detectable levels of 20E and JH
ED2	Embryo	48 h AO	12%	Abundant energids, germ band anlage well delimited, slightly expanded at both sides. Tanaka stage 2	Burst of 20E inferred from the expression of HR3 (a 20E–dependent gene). No detectable JH
ED6	Embryo	144 h AO	33%	Pleuropodia well apparent, legs segmented, caudal space arises. Tanaka stage 8	Peak of 20E. Very low levels of JH
ED13	Embryo	312 h AO	72%	Eyes well colored, antennae and legs reaching the 5th abdominal segment. Tanaka stage 15	Peak of 20E. High levels of JH
N1	1st nymphal instar	1–2 days old	**–**	**–**	High levels of 20E and JH
N3	3rd nymphal instar	2–4 days old	**–**	**–**	High levels of 20E and JH
N5	5th nymphal instar	3–5 days old	**–**	**–**	High levels of 20E and JH
N6	6th nymphal instar	5–7 days old	**–**	**–**	High levels of 20E, no JH
Adult	Adult (female)	5 days old	**–**	**–**	Low (ovarian) levels of 20E and high JH levels

**Fig. 1:**
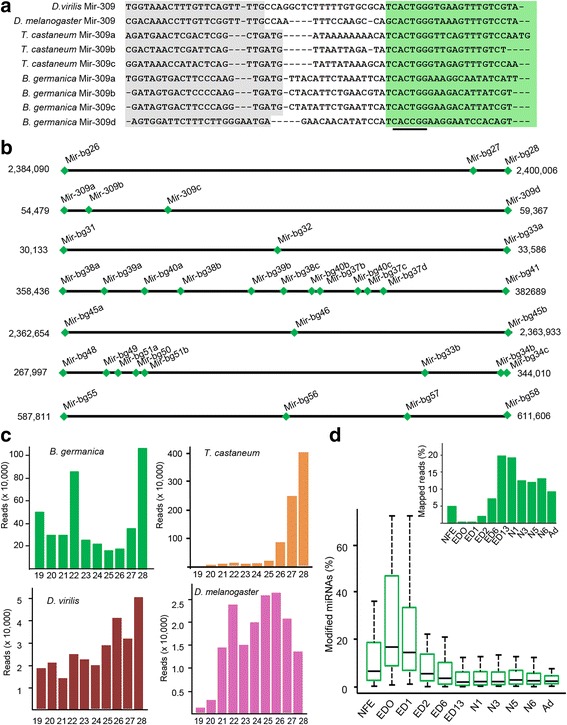
*B. germanica* miRNAs. **a** Precursor sequences of the MIR-309 miRNAs in *B. germanica*, *T. castaneum*, *D. melanogaster* and *D. virilis*. Highlighted in grey are the sequences corresponding to star miRNAs, and in green those corresponding to mature miRNAs. The conserved seed sequence is underlined. **b** Genomic clusters of newly identified *B. germanica* miRNAs containing more than 3 miRNA genes. c Read size distribution in the NFE small RNA libraries of the studied species; read length (abscissae) is expressed in number of nucleotides. d Frequency of miRNAs with 3-end modified reads in the developmental stages studied. The inset shows the relative abundance of miRNA reads per library. The abbreviations for the stages are explained in Table 1

Putative *B. germanica* miRNAs were then predicted using the mirDeep2 algorithm. The predictions included the 88 conserved insect miRNAs and 11 miRNAs previously discovered in *B. germanica* [10, 11], plus 264 novel miRNA candidates. After filtering the 264 candidates adhering to the biogenesis criteria [11, 20], 67 bona-fide *B. germanica* miRNA genes were obtained (Additional file 2: Table S2). Four of these belonged to the MIR-309 family (Fig. 1a), which is typical of insects [11] and that in *D. melanogaster* is involved in the clearance of maternally loaded mRNAs [21]. As no known orthologs of the other 63 miRNA genes were found, they were considered as newly discovered. Six of these genes contained between two and three identical, mature miRNAs sequences. Thus, 54 different novel mature miRNAs, grouped into 48 miRNA families, were finally identified as novel in *B. germanica* (Additional file 2: Table S2). These 54 miRNAs, plus the 11 previously reported [11], represent the full repertoire miRNAs only found in *B. germanica*. As seen for other *B. germanica* miRNAs [11], most of those reported herein for the first time grouped into genomic clusters, e.g., into the MIR-309 family (Fig. 1b). Moreover, 43 of the 65 miRNA genes now discovered in *B. germanica* grouped into nine additional clusters spanning 4–70 kb, and included 2–12 usually paralogous miRNA genes per cluster (Fig. 1b).

With respect to maternal miRNAs (non**-**fertilized eggs, NFE, library), the read length distribution (Fig. 1c) showed two prominent peaks, one at 22 nts corresponding to miRNAs, and the other at 28 nts that might correspond to Piwi-interacting RNAs (piRNAs) [16, 22]. Comparison of the NFE libraries of *T. castaneum*, *D. melanogaster* and *D. virilis* [16, 17] revealed the proportion of miRNAs and piRNAs to be different in *T. castaneum* (Fig. 1c), with the latter very much more abundant [17]. Interestingly, the *B. germanica* miRNAs in embryo day (ED) 0 and ED1 showed a higher frequency (15–26%) of reads with extra nucleotides at the 3′-end compared to other embryo or post-embryo stages (7–11%). Most of these 3′-end modifications were adenylations (approximately 50%) and uracylations (25%) (Fig. 1d). The peak of 3′-end modifications at ED0-ED1 coincide with the minimum relative number of miRNA reads (Fig. 1d, inset).

### Changes in miRNA expression over cockroach ontogeny

**Fig. 2:**
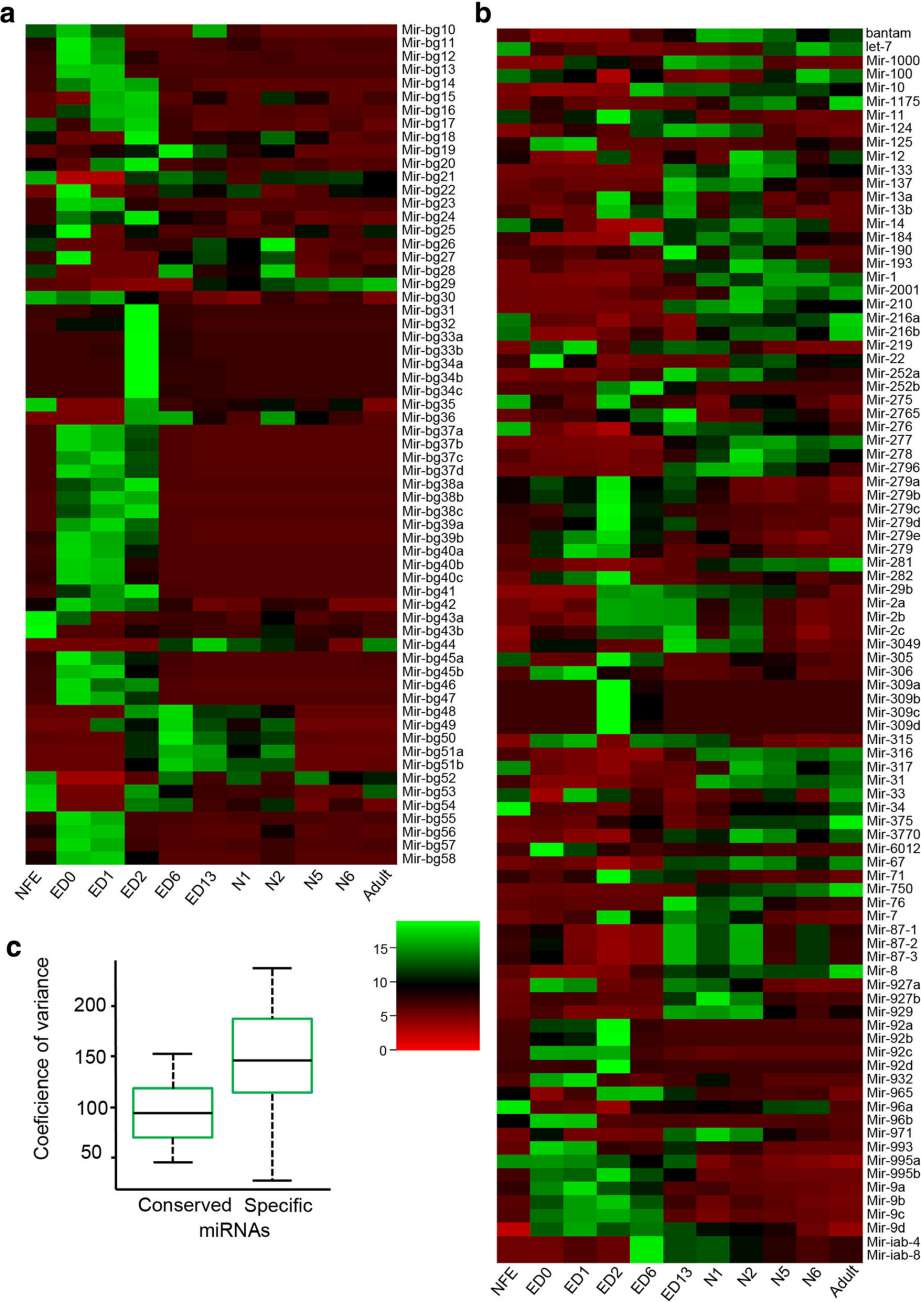
Expression of *B. germanica* miRNAs over ontogeny. a Heat map showing the expression of the newly found miRNAs. b Heat map showing the expression of the conserved miRNAs. The abbreviations of the stages are explained in Table 1. c Variation of the expression in conserved and specific miRNAs. The asterisk indicates statistically significant differences between the two samples (Welch’s t-test, *p* < 0.01)

**Fig. 3:**
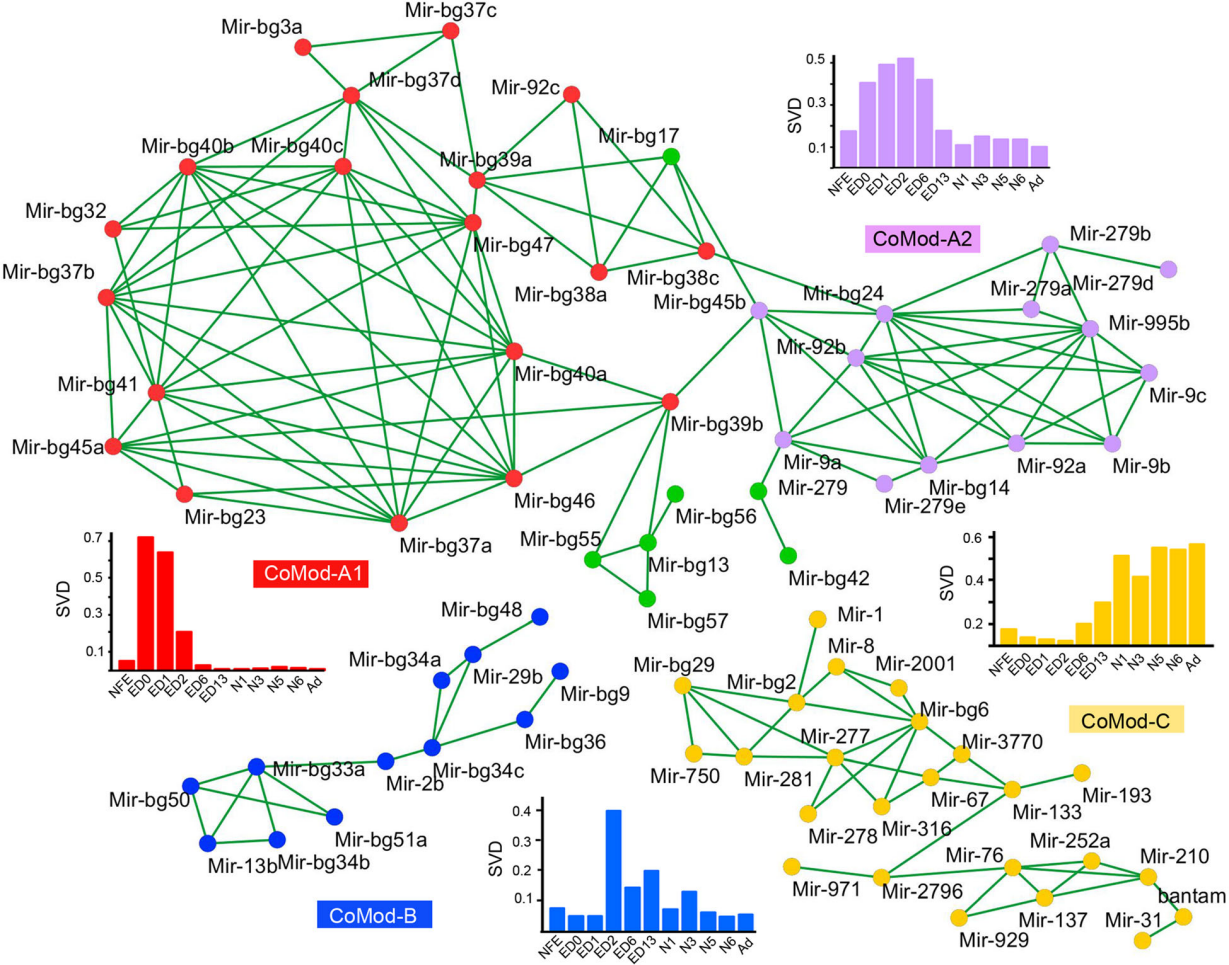
Coexpression modules of *B. germanica* miRNAs during ontogenesis. The expression patterns representing each module (metagene expression patterns) are shown besides the corresponding coexpression network

Most of the newly discovered miRNAs predominated in the embryo, especially in the early stages, from ED0 to ED2 (Fig. 2a, Additional file 3: Table S3). In contrast, conserved miRNAs tended to be expressed over a longer period of time (Fig. 2b, Additional file 3: Table S3). Therefore, the new and predominantly embryonic miRNAs showed a generally higher coefficient of variation in their expression over development (Fig. 2c, Additional file 4: Figure S1). The expression patterns of the 157 *B. germanica* miRNAs were very diverse (Fig. 2), but coexpression networks recognized through the use of Spearman correlation analysis allowed this diversity to be collapsed into four main co-expression modules (CoMod) (Fig. 3). CoMod-A grouped miRNAs generally expressed during embryonic development. In fact, CoMod-A can be subdivided into two sub-modules (Fig. 3): CoMod-A1, which groups miRNAs predominantly expressed during ED0-ED1, and CoMod-A2, which groups those mostly expressed between ED0 and ED6. CoMod-B is characterized by acute expression during ED2, thereafter decreasing in intensity. Finally, CoMod-C grouped miRNAs expressed during post-embryonic development. The respective metagene expression patterns of these four CoMods are shown in Fig. 3.

**Fig. 4:**
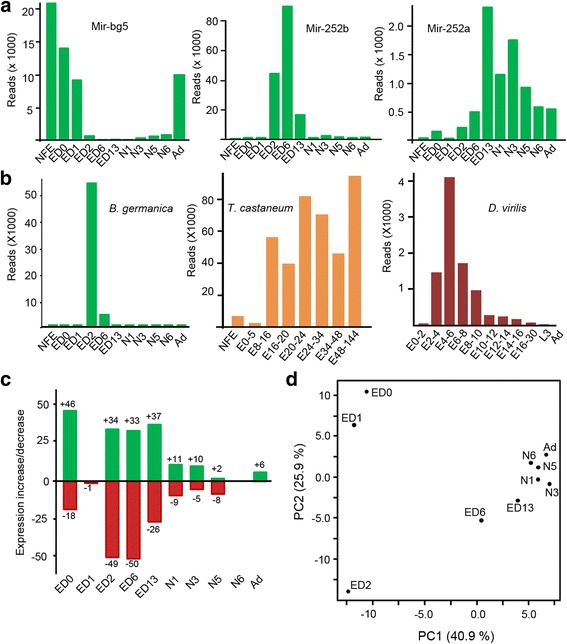
Changes of miRNAs expression over *B. germanica* ontogenesis. **a** Expression of Mir-bg5b, Mir-252b and Mir-252a. **b** Expression of MIR-309 miRNAs compared to that seen in *T. castaneum* and *D. virilis*. **c** Number of miRNA genes significantly (*p <* 0.05) upregulated (green) or downregulated (red) at each stage transition. **d** Principal component analysis plot based on miRNA expression in the embryo, nymph and adult stages. The abbreviations for the stages are explained in Table 1

Other, peculiar miRNA expression patterns were also seen. For example, Mir-bg5 was (strongly) expressed in very early embryos and in adult females (Fig. 4a). The comparison of Mir-252b and Mir-252a is interesting since both share the same seed sequence (although the corresponding genes are separated by some 40 kb in the genome). However, Mir-252b showed an expression peak during ED6 and a dramatic reduction in expression in ED13, while Mir-252a expression dramatically increased from ED13 onward (Fig. 4a). The expression of Mir-309 followed a CoMod-B pattern, showing an acute peak in ED2 (at 11% of development) (Fig. 4b). This is compatible with a possible role in the clearance of maternally loaded mRNAs, as seen in *D. melanogaster* [21]. The expression pattern of Mir-309 in *D. virilis* showed a peak at 13–20% of development (Fig. 4b), similar to that seen for *B. germanica*, whereas in *T. castaneum*, Mir-309 expression increased at 6–11% of development and then remained more or less stable until the end of embryogenesis (Fig. 4b).

Evidence of differential expression between stage-libraries was also sought. The transition between NFE and ED0 involved more changes than between ED1- ED13. The most conservative transitions were those between ED0 and ED1 (during which only one miRNA was downregulated), and between N5 and N6 (during which no changes were observed) (Fig. 4c, Additional file 5: Figure S2). Principal component analysis (PCA) of the expression data of all miRNAs in all libraries (Fig. 4d) highlighted the contrast between the compact group composed by nymphal and adult stages and that formed by the embryonic stages.

### Maternal and very early embryonic miRNAs in *Blattella*, *Tribolium* and *Drosophila*

**Fig. 5:**
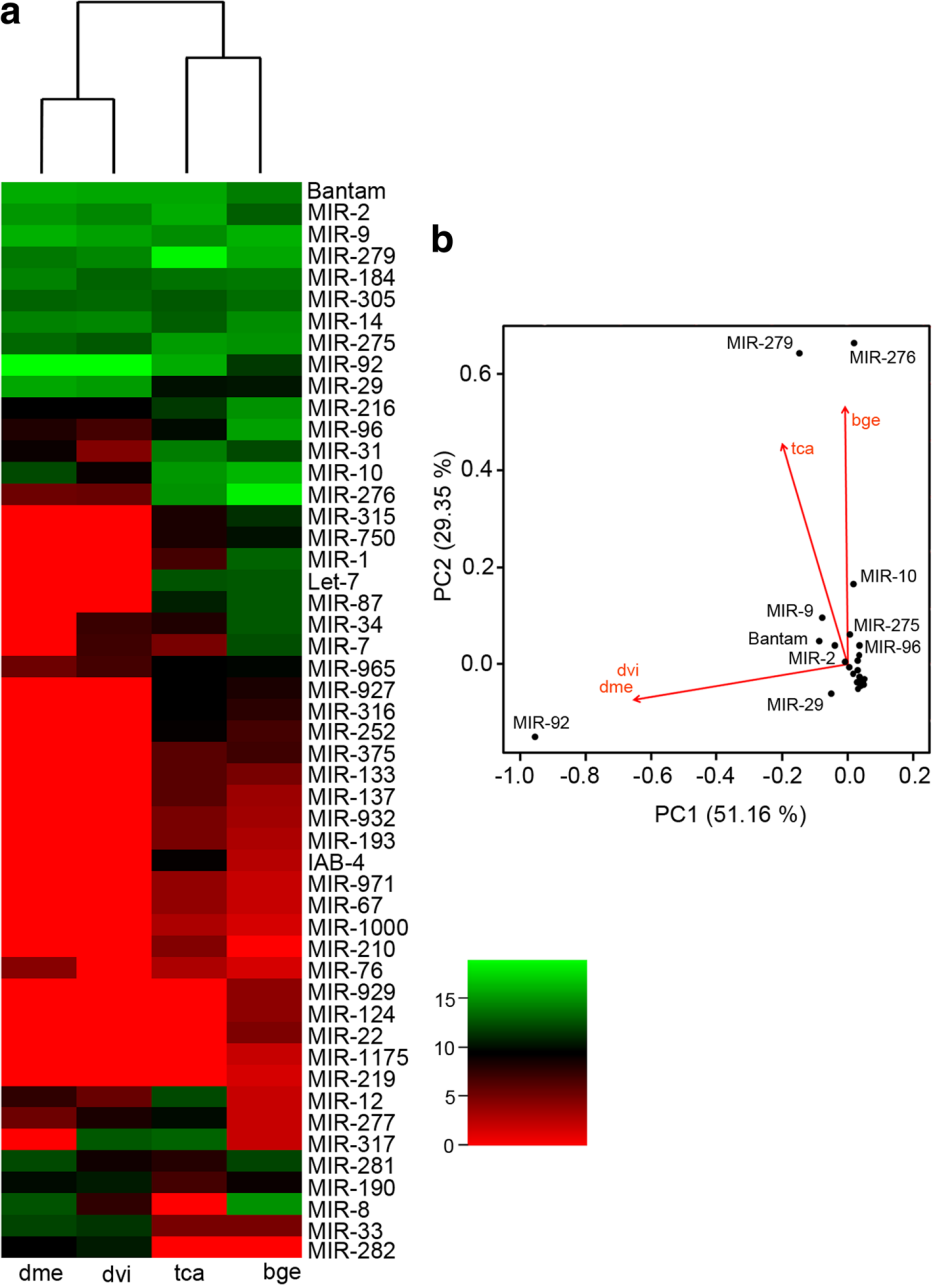
Families of conserved miRNAs in NFE for *B. germanica*, *T. castaneum*, *D. melanogaster* and *D. virilis*. **a** Heat map showing the relative abundance of miRNA families in each species and derived hierarchical clustering. **b** Principal component analysis plot showing the main drivers of the clustering. Bge: *B. germanica*, dme: *D. melanogaster*, dvi: *D. virilis* and tca: *T. castaneum*

To study the possible role of miRNAs in the definition of the germ-band type, the *B. germanica* NFE library (Table 1) was compared with the equivalent non-fertilized egg libraries of *T. castaneum*, *D. melanogaster* and *D. virilis* [16, 17]. Clustering analysis of the expression of orthologous miRNA families in the four species (Fig. 5a) separated the long germ-band species (*D. melanogaster* and *D. virilis*) from the two short germ-band species (*T. castaneum* and *B. germanica*). PCA (Fig. 5b) showed this grouping to be mostly driven by the MIR-276, MIR-279 and MIR-92 families. Though not highlighted by PCA, the miRNAs from the let-7 cluster were significantly expressed in *B. germanica* and *T. castaneum* NFE, but not in the *Drosophila* species studied. Further, the MIR-bg5 family was highly expressed in *B. germanica* (the fifth most abundant miRNA family), but not in *T. castaneum* or the *Drosophila* species.

**Fig. 6:**
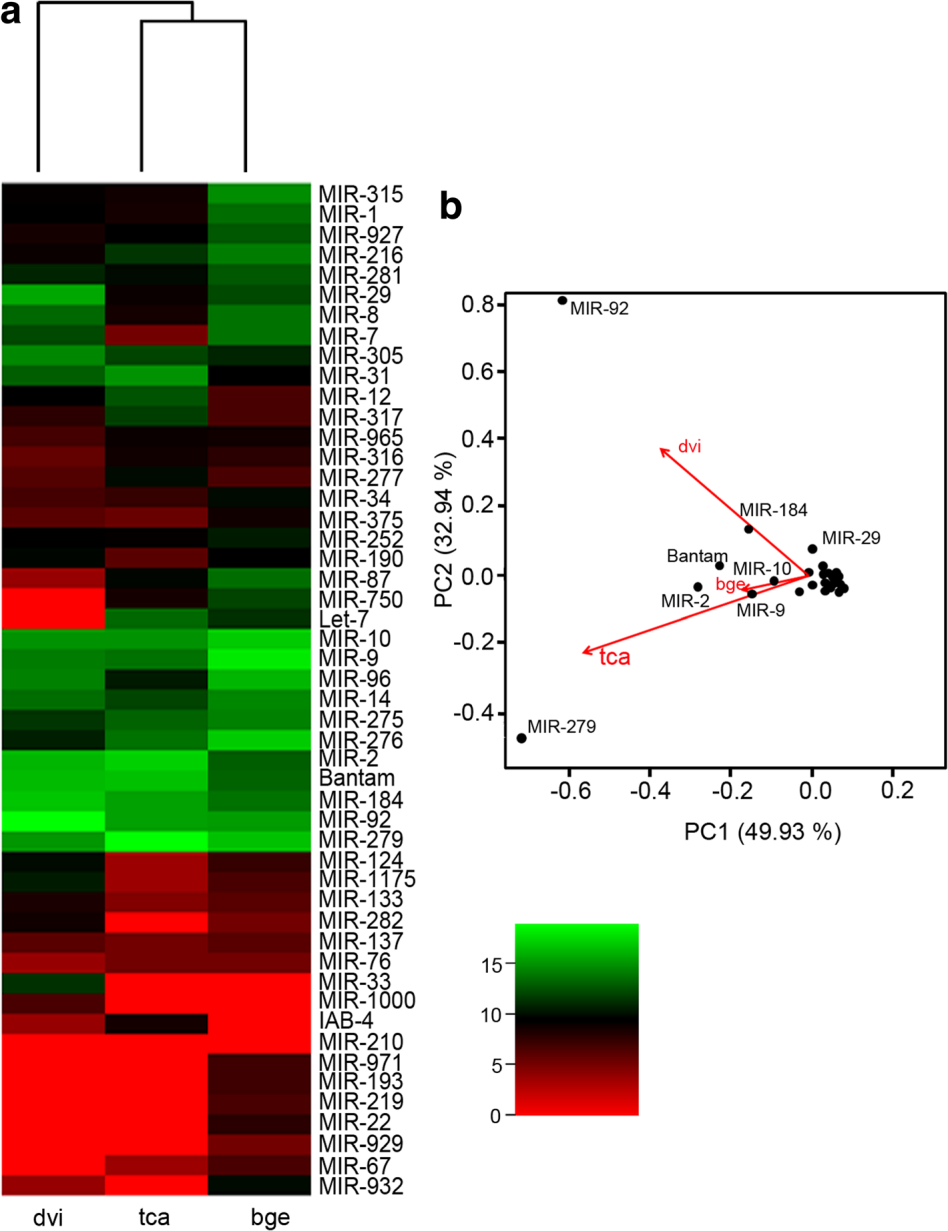
Families of conserved miRNAs in ED0 for *B. germanica*, *T. castaneum* and *D. virilis*. **a** Heat map representing the relative abundance of miRNA families in each species and derived hierarchical clustering. **b** Principal component analysis plot showing the main drivers of the clustering. The libraries were prepared at 1.85%, 0–3.5% and 0–6.67% of development in *B. germanica* (bge), *T. castaneum* (tca) and *D. virilis* (dvi), respectively

Comparisons were also made between *B. germanica* ED0 (8 h, 1.9% of development) library (Table 1) and the equivalent libraries for the first hours of embryonic development in *T. castaneum* (0**–**5 h, 0**–**3.5% of development) and *D. virilis* (0**–**2 h, 0**–**6.7% development) [16, 17]. In all three, the germ-band began to form at this time. The miRNA expression profiles were notably similar for the three species; indeed, clustering analysis hardly separated the long germ-band species (*D. virilis*) from the two short germ-band species (*T. castaneum* and *B. germanica*) (Fig. 6a). PCA showed the main drivers of this grouping to again be MIR-279 (for *B. germanica* and *T. castaneum*) and MIR-92 (for *D. virilis*) (Fig. 6b).

## Discussion

### The newly discovered miRNAs predominate in the embryo

The present results indicate the embryonic presence of the MIR-309 family of miRNAs in *B. germanica*, plus 54 novel miRNAs [10, 11]. *D. virilis* [16] and *T. castaneum* [17] have a high percentage of species-specific miRNAs during embryonic life. In the very early embryonic stages of *B. germanica*, the proportions of miRNAs and piRNAs are similar, resembling those observed in *D. melanogaster* and *D. virilis*. *T. castaneum*, in contrast, shows a high proportion of piRNAs in early ontogenesis [16, 17]. This is likely a peculiarity of the latter species, not attributable to either its germ-band type or mode of metamorphosis.

### The four main waves of miRNA expression during cockroach ontogenesis

The miRNA expression data showed that most miRNA changes occur in the embryo. This is consistent with the hemimetabolan mode of metamorphosis, in which the key developmental process leading to the adult body structure takes place in the embryo. In contrast, the nymphal transitions are developmentally conservative [19]. The transition from N5 to N6 appears to proceed without changes in the miRNAs, which is surprising since adult genetic program should be installed in N6. Also surprising is the small number of changes that occur in the transition from N6 to adult, when the adult program is executed [15, 23].

The maternal loading of NFE involves conserved miRNAs such as let-7, Bantam, Mir-34, Mir-305, Mir-8, Mir-71 and Mir-1, all of which play roles in basic biological functions [6], as well as large amounts of MIR-bg5 miRNAs. MIR-bg5 is specific for hemimetabolan species [11] and is highly abundant in NFE and in adult females of *B. germanica*. This, plus the observation that the expression of MIR-bg5 miRNAs occur in the ovaries of adult females (see [10], where Mir-bg5 is called “Novel 1”), indicates that members of this family are typically maternally loaded miRNAs, at least in *B. germanica*.

The first two waves of miRNA expression, represented by CoMod-A1 and CoMod-A2, involve Mir-279, which is associated with multiple biological processes [24], and let-7 and Mir-9, which are related with neural differentiation and function [25–27]. A third wave (CoMod-B) that peaks during ED2, contains MIR-2 miRNAs, which are related to cell differentiation, development, morphogenesis and neurogenesis [28]. This wave also involves Mir-29, which is associated to neural cell development [26]. The final wave, represented by CoMod-C, is expressed in late embryos and post-embryonic development and involves bantam, Mir-1, Mir-8, Mir-278, Mir- 281, Mir-252 and Mir-31. These miRNAs are also among the best expressed of larval transcriptomes in *D. virilis* [16]. The most well-known is bantam, which is associated to basic functions in practically all stages of development [6]. In *D. melanogaster* larvae, Mir-8 has been related to the regulation of growth factors in body fat [29] and with body size [30], whereas Mir-278 and Mir-277 influence energy homeostasis [31, 32]. In *B. germanica* nymphs, Mir-8 regulates atrophin, a factor contributing to neuromotor coordination [14], whereas Mir-252 is involved in the regulation of general growth [13].

### The maternal-to-zygotic transition (MZT)

Degradation and clearance of maternal miRNAs is a key step in the MZT [33, 34], and adenylation at the 3′ end of maternal miRNAs has been shown to act as degradation signal. This modification has been observed in the fruit fly, sea urchin and mouse, which indicates that it is evolutionarily conserved [33]. Most miRNAs expressed between NFE and ED1 were highly adenylated at the 3′ end. These included the MIR-92 and MIR-10 families, which are also highly adenylated in early embryos of *T. castaneum* [17]. In *B. germanica*, the proportion of adenylated miRNAs peaked at ED0-ED1 when the relative amount of miRNAs is at its lowest. Adenylation therefore appears to contribute towards the clearing of maternally deposited miRNAs in *B. germanica* as well.

Although miRNAs are not the only players involved in the degradation of maternal RNAs, they play an important role in this regard. Indeed, our results suggest that a number of early embryo miRNAs might contribute towards clearing maternally loaded mRNAs. In zebrafish, the Mir-430 cluster of miRNAs is key for clearing maternal mRNA in the MZT [35]. In *D. melanogaster*, maternal mRNA clearing has been associated with the Mir-309 cluster, which includes Mir-9/4/79, Mir-5/6/2944, Mir-3/309 and Mir-279/286, which are mainly expressed between 9 and 12% of embryo development [21]. In *T. castaneum*, these miRNA families also appear involved in regulating maternally deposited mRNAs, along with several additional miRNA families that have been only found in *T. castaneum*, which are expressed between 6 and 11% of development [17]. In *B. germanica*, an acute expression peak of the Mir-309 cluster miRNAs (which comprises four Mir-309 paralogs) is observed at ED2 (11% of development), which might be involved in maternal mRNA clearance. The miRNAs of the CoMod-B peak at ED2 as well, and might also be involved in maternal mRNA clearance. Finally, Mir-9 and Mir-279, which are associated with maternal mRNA clearance in *D. melanogaster* [21], belong to CoMod-A2 and show high levels of expression in ED2. Taken together, these data suggest that maternal RNA clearing would involve two successive processes, the first (taking place around ED0-ED1), in which maternal adenylated miRNAs would be degraded, and the second (in ED2), in which maternal mRNAs would be cleared by the activity of Mir-309 and associated scavenger miRNAs.

### miRNAs, germ band and metamorphosis

Our analyses in the very early stages of embryo development (NFE and ED0) of *B. germanica*, *T. castaneum*, *D. melanogaster* and *D. virilis* indicated that miRNAs from MIR-276 and MIR-279 families are differentially expressed in short germ-band species, whereas those of MIR-92 family are differentially expressed in long germ-band species. In *D. melanogaster*, MIR-279 has been related to maternal mRNA clearance in the MZT transition (see above), restricting the emergence of CO_2_-sensing neurons, the maintenance of circadian rhythm, and the regulation of ovarian border cells [24]. The only reported function for MIR-276 is to promote egg-hatching synchrony by upregulating the transcription coactivator gene *brahma* in the locust *Locusta migratoria* [36]. MIR-92 miRNAs downregulate *jigr1* in the larval brain of *D. melanogaster*, which is essential for maintaining neuroblast self-renewal [25]. In the tobacco hawk moth *Manduca sexta*, Mir-92 is clearly overexpressed in embryos compared to larvae, pupae and adults [37]. Mir-92 is expressed in ED0 and ED1 of *B. germanica*, being one of the miRNAs highly adenylated at the 3′ end, which suggests that it is cleared in the MZT. If so, then its function could be restricted to very early embryogenesis, possibly to the formation of the germ-band. At NFE and ED0, the let-7 cluster is well expressed in *B. germanica* and *T. castaneum* but not in *Drosophila* spp. The let-7 cluster has traditionally been thought involved in the regulation of developmental transitions [38].

Clustering analyses of *B. germanica*, *T. castaneum* and the *Drosophila* spp. very early embryonic stage libraries did not separate the hemimetabolan and holometabolan species. Differences between these modes of metamorphosis must, however, exist, although they are perhaps clearer in more advanced stages of development, when the body structure is built. MIR-bg5 miRNAs have only been identified in hemimetabolan insects, and have been assumed lost in holometabolans [11, 39]. In *B. germanica*, they are maternally loaded in high amounts, which indicate them to operate in the early stages of embryo development, thus suggesting that MIR-bg5 miRNAs contribute towards determining the formation of the adult body plan characteristic of hemimetabolan even at the very early stages of embryogenesis.

## Conclusions

The present results show that miRNAs follow well-defined patterns of expression over hemimetabolan ontogeny, patterns that are more diverse during embryonic development than during the nymphal stages. This comes as no surprise since in hemimetabolan species most of the morphogenetic processes are concentrated into the embryonic stages, whereas nymphal development is morphogenetically conservative. The results also suggest that miRNAs play important roles in the developmental transitions between the embryonic stages of development (starting with maternal loading), during which they might influence the maternal-to-zygotic transition, the germ-band type and the metamorphosis mode.

## Methods

### Insects

*B. germanica* specimens were obtained from a colony reared in the dark at 29 ± 1 °C and 60–70% relative humidity. All dissections and tissue sampling procedures were performed on carbon dioxide-anesthetized specimens. Tissues were frozen in liquid nitrogen and stored at −80 °C until use.

### *B. germanica* Libraries of small RNAs

Eleven developmental stages were selected for analysis (Table 1). Data on juvenile hormone (JH) and ecdysteroids (20E) for the chosen stages are from Treiblmayr et al. [40] (JH in nymphal stages), Maestro et al. [41] (JH in embryo stages), Cruz et al. [42] (20E in nymphal stages), Piulachs et al. [43] (20E in embryo stages). Tanaka stages are from Tanaka [44]. For each stage, two small RNA libraries were prepared using the Illumina NEBNext small RNA libraries kit. These libraries were sequenced at the Pompeu Fabra University (Barcelona) Genomic Core Facility using the Illumina NextSeq platform (two runs paired end × 50 cycles).

### miRNA nomenclature

In general, the miRNA nomenclature system proposed by Fromm et al. [20] was followed. For the novel *B. germanica* miRNAs reported here for the first time, the system proposed by Ylla et al. [11] was used; this employs as designator name the code “bg” followed by a number assigned to the novel miRNA.

### miRNA predictions

Low quality reads were filtered out and adapters removed for the 22 small RNA-Seq libraries of *B. germanica* using Trimmomatic software [45]*.* Read pairs were then merged using the Pear tool [46]. The resulting reads longer than 18 nts were mapped to the *B. germanica* genome assembly [https://www.hgsc.bcm.edu/arthropods/german-cockroach-genome-project] using Bowtie2 software [47]. The files containing the reads aligned to the genome were used as input for the mirDeep2 miRNA prediction program. Novel miRNA candidates predicted by mirDeep2 [48] were submitted to mirPlot and filtered according to miRNA biogenesis criteria as previously described [11, 20]. Candidates passing all filtering criteria were considered novel miRNAs; these were then grouped into families on the basis of their seed sequence.

### Quantification of miRNA expression

Small RNA-Seq data for holometabolan insects were obtained from the literature [16, 17]. These included 10 small RNA-Seq libraries for eight stages of *T. castaneum* embryo development (GSE63770), 21 small RNA-Seq libraries for 12 stages of *Drosophila virilis* development (GSE54009 & GSE63770), and two libraries for non-fertilized eggs of *D. melanogaster* (GSE63770). Four additional libraries for two embryonic stages of *D. melanogaster* were obtained from modEncode [49]. Read adapters were removed using the fastx_clipper algorithm from the fast-toolkit at http://hannonlab.cshl.edu/fastx_toolkit/commandline.htmlt. The read fraction between 16 and 29 nts was then selected. These reads were mapped to the corresponding insect genomes: *T. castaneum* (tca_v4), *D. melanogaster* (NCBI_build5) and *D. virilis* (dvir_r1). The number of reads mapping to each annotated miRNA was determined using the FeatureCounts R package [50]. miRNA annotations for the *Drosophila* species were retrieved from the miRBase v.21 [51]. For *T. castaneum*, the miRBase miRNAs were complemented with the 123 miRNAs described by Ninova and collaborators [17]. For *B. germanica* those previously published were used [11] plus the novel miRNAs described in this paper. Finally, the miRNA counts were normalized as counts per million (CPM) with the edgeR package [52]. For comparing miRNA expression across insects, the miRNA genes were grouped into families as described previously [11]. Only orthologous families present in all compared insects were considered. The expression of each miRNA family was computed as the sum of the CPM corresponding to the miRNA members of that family. 3′-end modifications of *B. germanica* miRNAs were further assessed by obtaining those small RNA-Seq reads mapping to an annotated miRNA and containing nucleotides surpassing the 3′-end boundaries of the miRNA annotation.

### Differential expression analysis of miRNAs

The miRNA expression changes between one stage and the next were determined using the normalization and differential expression functions of the DESeq2 R package [53]. Only those miRNAs with a *p*-value adjusted for a false discovery rate of <0.05 were deemed to have a significant change of expression.

### Coexpression networks

The miRNA expression correlation between each pair of miRNA genes was determined using the Spearman correlation coefficient test; this returns the best performance in modeling RNA-Seq coexpression networks [54]. A pair of miRNAs are considered to be connected in the network if their correlation coefficient is higher than 0.9. The largest module was then divided into submodules, applying a correlation coefficient cut-off of 0.925. The characteristic expression profile of each module and submodule was determined using singular value decomposition [55–57].

## Additional files

Additional file 1: Table S1. Reads obtained from the small RNA libraries sequenced in *Blattella germanica*. (PDF 18 kb)

Additional file 2: Table S2. Description of the 67 miRNAs newly identified in *Blattella germanica*. (XLS 41 kb)

Additional file 3: Table S3. Read counts of each miRNA gene in the *Blattella germanica* stage-libraries studied. (XLS 56 kb)

Additional file 4: Figure S1. Coefficient of variation of the expression of newly found and conserved miRNAs during *Blattella germanica* development. (PDF 10623 kb)

Additional file 5: Figure S2. *P*-values of the differential expression analysis of the miRNA genes at each stage transition during *Blattella germanica* development. (PDF 4965 kb)

## Abbreviations

CoMod: Co-expression module
CPM: Counts per million
ED: Embryo day (0, 1, 2, 6, 13)
kb: Kilobase
miRNA: MicroRNA
MZT: Maternal-to-zygotic transition
N: Nymph (1, 3, 5, 6)
NFE: Non-fertilized eggs
nts: Nucleotides
PCA: Principal component analysis
piRNA: Piwi-interacting RNA

## References

[1] Chipman AD (2015). Hexapoda: comparative aspects of early development. Evolutionary developmental biology of invertebrates.

[2] Peel AD, Chipman AD, Akam M (2005). Arthropod segmentation: beyond the *Drosophila* paradigm. Nat Rev Genet.

[3] Lynch JA, El-Sherif E, Brown SJ (2012). Comparisons of the embryonic development of *Drosophila*, *Nasonia* and *Tribolium*. Wiley Interdiscip Rev Dev Biol.

[4] Liu PZ, Kaufman TC (2005). Short and long germ segmentation: unanswered questions in the evolution of a developmental mode. Evol Dev.

[5] Bushati N, Cohen SM (2007). microRNA functions. Annu Rev Cell Dev Biol.

[6] Belles X, Cristino AS, Tanaka ED, Rubio M, Piulachs M-D, Gilbert LI (2012). Insect MicroRNAs: from molecular mechanisms to biological roles. Insect molecular biology and biochemistry.

[7] Flynt AS, Lai EC (2008). Biological principles of microRNA-mediated regulation: shared themes amid diversity. Nat Rev Genet.

[8] Ebert MS, Sharp PA (2012). Roles for MicroRNAs in conferring robustness to biological processes. Cell.

[9] Hornstein E, Shomron N (2006). Canalization of development by microRNAs. Nat Genet.

[10] Cristino AS, Tanaka ED, Rubio M, Piulachs M-D, Belles X. Deep sequencing of organ- and stage-specific micrornas in the evolutionarily basal insect *Blattella germanica* (L.) (Dictyoptera, Blattellidae). PLOS One. 2011;6. 10.1371/journal.pone.0019350 PMC3084283 21552535

[11] Ylla G, Fromm B, Piulachs M-D, Belles X (2016). The microRNA toolkit of insects. Sci Rep.

[12] Rubio M, Belles X (2013). Subtle roles of microRNAs let-7, miR-100 and miR-125 on wing morphogenesis in hemimetabolan metamorphosis. J Insect Physiol.

[13] Rubio M, de Horna A, Belles X (2012). MicroRNAs in metamorphic and non-metamorphic transitions in hemimetabolan insect metamorphosis. BMC Genomics.

[14] Rubio M, Montañez R, Perez L, Milan M, Belles X (2013). Regulation of atrophin by both strands of the mir-8 precursor. Insect Biochem Mol Biol.

[15] Lozano J, Montañez R, Belles X (2015). MiR-2 family regulates insect metamorphosis by controlling the juvenile hormone signaling pathway. Proc Natl Acad Sci U S A.

[16] Ninova M, Ronshaugen M, Griffiths-Jones S (2014). Conserved temporal patterns of MicroRNA expression in *Drosophila* support a developmental hourglass model. Genome Biol Evol.

[17] Ninova M, Ronshaugen M, Griffiths-Jones S (2016). MicroRNA evolution, expression, and function during short germband development in *Tribolium castaneum*. Genome Res.

[18] Truman JW, Riddiford LM (1999). The origins of insect metamorphosis. Nature.

[19] Belles X (2011). Origin and evolution of insect metamorphosis. eLS.

[20] Fromm B, Billipp T, Peck LE, Johansen M, Tarver JE, King BL (2015). A uniform system for the annotation of vertebrate microRNA genes and the evolution of the human microRNAome. Annu Rev Genet.

[21] Bushati N, Stark A, Brennecke J, Cohen SM (2008). Temporal reciprocity of miRNAs and their targets during the maternal-to-zygotic transition in *Drosophila*. Curr Biol.

[22] Aravin A, Gaidatzis D, Pfeffer S, Lagos-Quintana M, Landgraf P, Iovino N (2006). A novel class of small RNAs bind to MILI protein in mouse testes. Nature.

[23] Belles X, Santos CG (2014). The MEKRE93 (Methoprene tolerant-Krüppel homolog 1-E93) pathway in the regulation of insect metamorphosis, and the homology of the pupal stage. Insect Biochem Mol Biol.

[24] Sun K, Jee D, de Navas LF, Duan H, Lai EC (2015). Multiple in vivo biological processes are mediated by functionally redundant activities of *Drosophila* mir-279 and mir-996. PLoS Genet.

[25] Yuva-Aydemir Y, X-L X, Aydemir O, Gascon E, Sayin S, Zhou W (2015). Downregulation of the host gene jigr1 by miR-92 is essential for neuroblast self-renewal in drosophila. PLoS Genet.

[26] Smirnova L, Gräfe A, Seiler A, Schumacher S, Nitsch R, Wulczyn FG (2005). Regulation of miRNA expression during neural cell specification. Eur J Neurosci.

[27] Coolen M, Bally-Cuif L (2009). MicroRNAs in brain development and physiology. Curr Opin Neurobiol.

[28] Marco A, Hooks K, Griffiths-Jones S (2012). Evolution and function of the extended miR-2 microRNA family. RNA Biol.

[29] Lee GJ, Jun JW, Hyun S (2015). MicroRNA miR-8 regulates multiple growth factor hormones produced from *Drosophila* fat cells. Insect Mol Biol.

[30] Jin H, Kim VN, Hyun S (2012). Conserved microRNA miR-8 controls body size in response to steroid signaling in *Drosophila*. Genes Dev.

[31] Esslinger SM, Schwalb B, Helfer S, Michalik KM, Witte H, Maier KC (2013). *Drosophila* miR-277 controls branched-chain amino acid catabolism and affects lifespan. RNA Biol.

[32] Teleman AA, Maitra S, Cohen SM (2006). *Drosophila* lacking microRNA miR-278 are defective in energy homeostasis. Genes Dev.

[33] Lee M, Choi Y, Kim K, Jin H, Lim J, Nguyen TA (2014). Adenylation of maternally inherited microRNAs by wispy. Mol Cell.

[34] Marco A (2015). Selection against maternal microRNA target sites in maternal transcripts. G3 (Bethesda).

[35] Giraldez AJ, Mishima Y, Rihel J, Grocock RJ, Van Dongen S, Inoue K (2006). Zebrafish MiR-430 promotes deadenylation and clearance of maternal mRNAs. Science.

[36] He J, Chen Q, Wei Y, Jiang F, Yang M, Hao S (2016). MicroRNA-276 promotes egg-hatching synchrony by up-regulating *brm* in locusts. Proc Natl Acad Sci U S A.

[37] Zhang X, Zheng Y, Jagadeeswaran G, Ren R, Sunkar R, Jiang H (2012). Identification and developmental profiling of conserved and novel microRNAs in *Manduca sexta*. Insect Biochem Mol Biol.

[38] Faunes F, Larraín J (2016). Conservation in the involvement of heterochronic genes and hormones during developmental transitions. Dev Biol.

[39] Belles X (2017). MicroRNAs and the evolution of insect metamorphosis. Annu Rev Entomol.

[40] Treiblmayr K, Pascual N, Piulachs M-D, Keller T, Belles X. Juvenile hormone titer versus juvenile hormone synthesis in female nymphs and adults of the German cockroach, Blattella germanica. J Insect Sci. 2006;6:1–7. 10.1673/031.006.4301 PMC2990300 20233097

[41] Maestro JL, Pascual N, Treiblmayr K, Lozano J, Belles X. Juvenile hormone and allatostatins in the German cockroach embryo. Insect Biochem Mol Biol. 2010;40:660–5. 10.1016/j.ibmb.2010.06.006 20542115

[42] Cruz J, Martín D, Pascual N, Maestro JL, Piulachs MD, Belles X. Quantity does matter. Juvenile hormone and the onset of vitellogenesis in the German cockroach. Insect Biochem Mol Biol. 2003;33:1219–25. 10.1016/j.ibmb.2003.06.004 14599494

[43] Piulachs M-D, Pagone V, Belles X. Key roles of the Broad-Complex gene in insect embryogenesis. Insect Biochem Mol Biol. 2010;40:468–75. 10.1016/j.ibmb.2010.04.006 20403438

[44] Tanaka A. Stages in the embryonic development of the German cockroach, Blattella germanica Linné (Blattaria, Blattellidae). Kontyû, Tokyo. 1976;44:1703–14.

[45] Bolger AM, Lohse M, Usadel B (2014). Trimmomatic: a flexible trimmer for Illumina sequence data. Bioinformatics.

[46] Zhang J, Kobert K, Flouri T, Stamatakis A (2014). PEAR: a fast and accurate Illumina paired-end reAd mergeR. Bioinformatics.

[47] Langmead B, Salzberg SL (2012). Fast gapped-read alignment with bowtie 2. Nat Methods.

[48] Friedländer MR, Mackowiak SD, Li N, Chen W, Rajewsky N (2012). miRDeep2 accurately identifies known and hundreds of novel microRNA genes in seven animal clades. Nucleic Acids Res.

[49] Celniker SE, Dillon LA, Gerstein MB, Gunsalus KC, Henikoff S, Karpen GH (2009). Unlocking the secrets of the genome. Nature.

[50] Liao Y, Smyth GK, Shi W (2014). featureCounts: an efficient general purpose program for assigning sequence reads to genomic features. Bioinformatics.

[51] Kozomara A, Griffiths-Jones S (2011). miRBase: integrating microRNA annotation and deep-sequencing data. Nucleic Acids Res.

[52] Robinson MD, McCarthy DJ, Smyth GK (2010). edgeR: a bioconductor package for differential expression analysis of digital gene expression data. Bioinformatics.

[53] Love MI, Huber W, Anders S (2014). Moderated estimation of fold change and dispersion for RNA-seq data with DESeq2. Genome Biol.

[54] Ballouz S, Verleyen W, Gillis J (2015). Guidance for RNA-seq co-expression network construction and analysis: safety in numbers. Bioinformatics.

[55] Golub GH, Van LCF (1996). Matrix computations.

[56] Tomfohr J, Lu J, Kepler TB (2005). Pathway level analysis of gene expression using singular value decomposition. BMC Bioinforma.

[57] Alter O, Brown PO, Botstein D (2000). Singular value decomposition for genome-wide expression data processing and modeling. Proc Natl Acad Sci U S A.

