# Supplementary material for: Comparative analysis of miRNA expression during the development of insects of different metamorphosis modes and germ-band types
Source: BMC Genomics. 2017 Oct 11;18:774. doi: 10.1186/s12864-017-4177-5 (PMC5637074; doi:10.1186/s12864-017-4177-5)
Supplement: Supplementary file 1 — Reads obtained from the small RNA libraries sequenced in Blattella germanica. (PDF 18 kb) [file 12864_2017_4177_MOESM1_ESM.pdf]

Table S1. Reads obtained from the small RNA libraries sequenced in *Blattella germanica*. Number of raw reads, clean reads after Pear and Trimmomatic filtering and number of mapped reads to the genome for each library

**Library-replicate**	**Raw reads**	Clean Reads	**% Clean reads**	**Mapped reads**
NFE_1	15.188.160	10.362.275	68,23%	8.216.901
NFE_2	10.477.701	5.112.361	48,79%	4.114.970
ED0_1	14.988.607	8.251.659	55,05%	6.420.620
ED0_2	13.328.184	7.205.895	54,07%	5.784.482
ED1_1	17.013.482	10.406.061	61,16%	8.221.343
ED1_2	13.626.544	8.595.023	63,08%	6.979.270
ED2_1	15.544.081	11.376.197	73,19%	9.341.651
ED2_2	9.196.837	5.369.022	58,38%	4.580.778
ED6_1	11.514.721	4.966.561	43,13%	4.600.004
ED6_2	9.767.761	5.512.509	56,44%	4.923.115
ED13_1	15.702.288	11.883.929	75,68%	10.769.918
ED13_2	15.869.277	10.850.606	68,37%	9.690.882
N1_1	16.944.588	12.615.221	74,45%	10.564.079
N1_2	10.721.221	6.588.109	61,45%	5.583.152
N3_1	15.701.436	9.229.812	58,78%	7.946.229
N3_2	11.506.488	6.843.707	59,48%	5.913.912
N5_1	14.138.682	7.433.964	52,58%	6.204.393
N5_2	10.783.752	6.154.281	57,07%	5.119.692
N6_1	15.091.392	9.173.526	60,79%	7.837.241
N6_2	10.002.460	6.417.770	64,16%	5.375.478
Adult_1	14.994.421	8.847.418	59,00%	7.093.111
Adult_2	9.776.210	4.391.990	44,93%	3.393.035
