# Supplementary material for: Comparative analysis of miRNA expression during the development of insects of different metamorphosis modes and germ-band types
Source: BMC Genomics. 2017 Oct 11;18:774. doi: 10.1186/s12864-017-4177-5 (PMC5637074; doi:10.1186/s12864-017-4177-5)
Supplement: Supplementary file 4 — Coefficient of variation of the expression of newly found and conserved miRNAs during Blattella germanica development. (PDF 10623 kb) [file 12864_2017_4177_MOESM4_ESM.pdf]

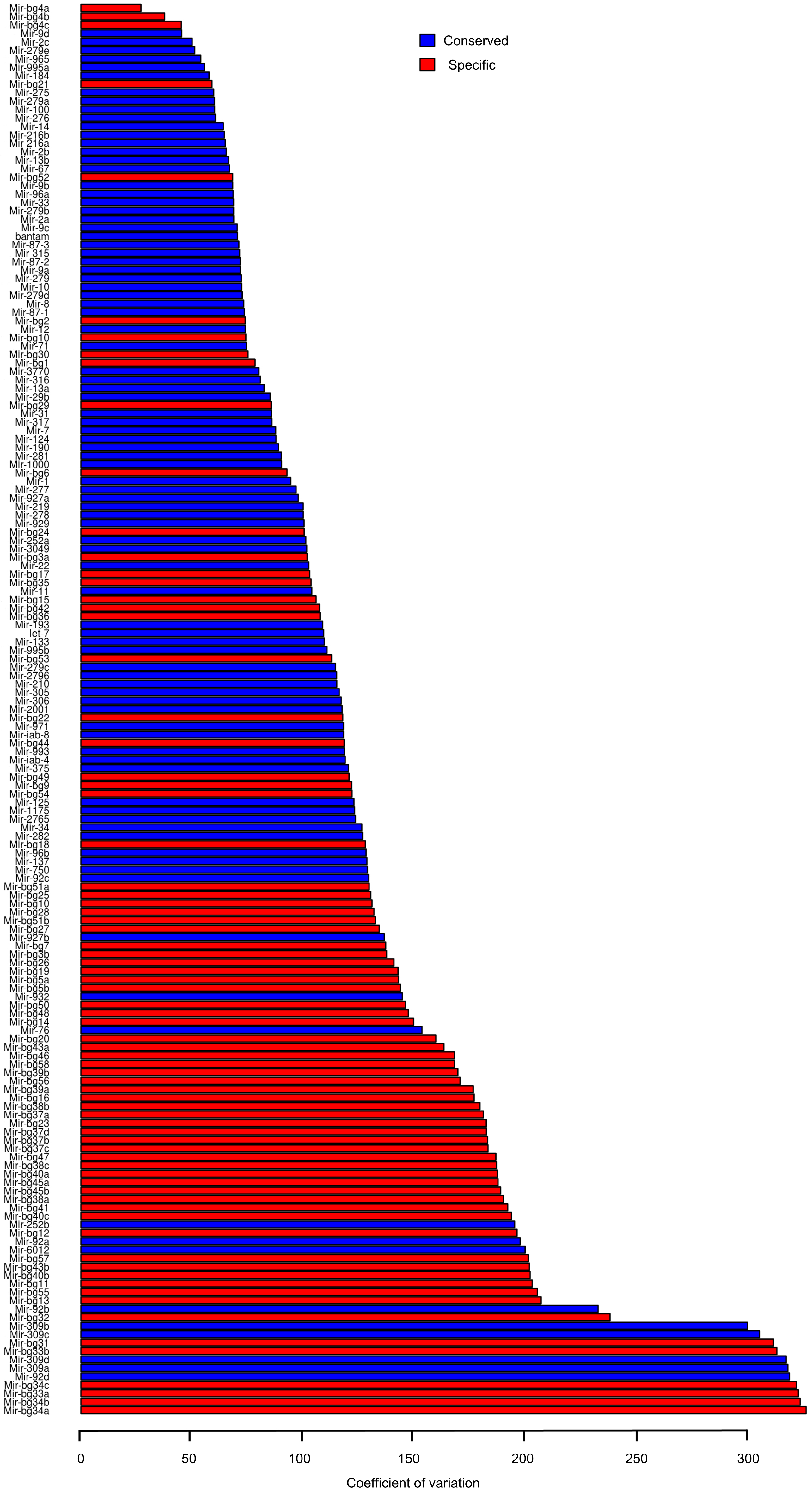

Fig. S1. Coefficient of variation of the expression of specific and conserved miRNAs during *Blattella germanica* development.
