# Supplementary material for: Comparative analysis of miRNA expression during the development of insects of different metamorphosis modes and germ-band types
Source: BMC Genomics. 2017 Oct 11;18:774. doi: 10.1186/s12864-017-4177-5 (PMC5637074; doi:10.1186/s12864-017-4177-5)
Supplement: Supplementary file 5 — P-values of the differential expression analysis of the miRNA genes at each stage transition during Blattella germanica development. (PDF 4965 kb) [file 12864_2017_4177_MOESM5_ESM.pdf]

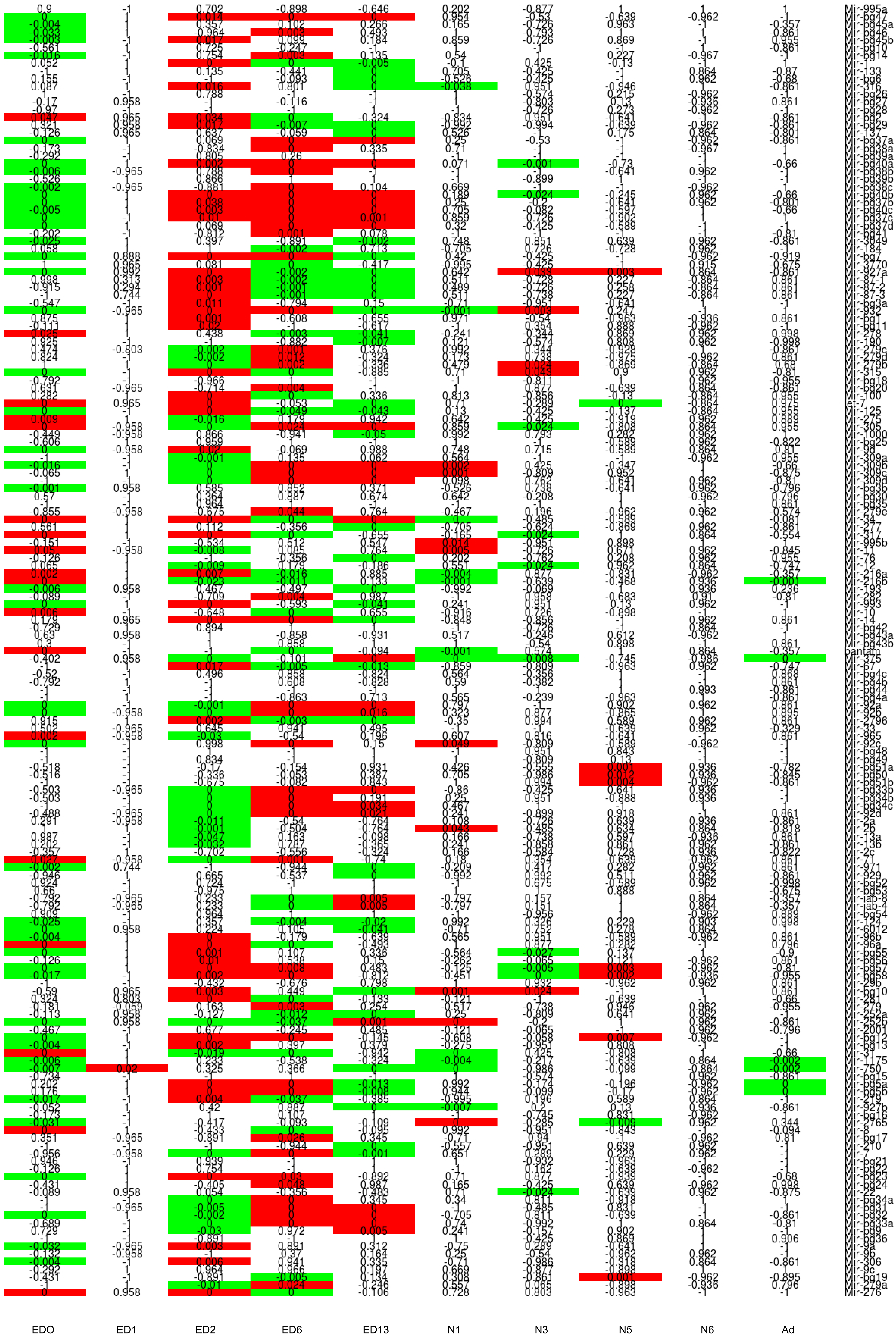

Fig. S2. P-values of the differential expression analysis of the miRNA genes at each stage transition during *Blattella germanica* development. Significant pvalues (<0.05) are highlighted in green or red, indicating that miRNA expression is upregulated or downregulated, respectively.
